# Discriminating Suicide Attempters and Predicting Suicide Risk Using Altered Frontolimbic Resting-State Functional Connectivity in Patients With Bipolar II Disorder

**DOI:** 10.3389/fpsyt.2020.597770

**Published:** 2020-11-26

**Authors:** Rongxin Zhu, Shui Tian, Huan Wang, Haiteng Jiang, Xinyi Wang, Junneng Shao, Qiang Wang, Rui Yan, Shiwan Tao, Haiyan Liu, Zhijian Yao, Qing Lu

**Affiliations:** ^1^Department of Psychiatry, The Affiliated Brain Hospital of Nanjing Medical University, Nanjing, China; ^2^School of Biological Sciences and Medical Engineering, Southeast University, Nanjing, China; ^3^Child Development and Learning Science, Key Laboratory of Ministry of Education, Southeast University, Nanjing, China; ^4^Nanjing Brain Hospital, Medical School of Nanjing University, Nanjing, China

**Keywords:** bipolar II disorder, resting-state functional connectivity, frontolimbic system, suicide attempt, support vector machine

## Abstract

Bipolar II disorder (BD-II) major depression episode is highly associated with suicidality, and objective neural biomarkers could be key elements to assist in early prevention and intervention. This study aimed to integrate altered brain functionality in the frontolimbic system and machine learning techniques to classify suicidal BD-II patients and predict suicidality risk at the individual level. A cohort of 169 participants were enrolled, including 43 BD-II depression patients with at least one suicide attempt during a current depressive episode (SA), 62 BD-II depression patients without a history of attempted suicide (NSA), and 64 demographically matched healthy controls (HCs). We compared resting-state functional connectivity (rsFC) in the frontolimbic system among the three groups and explored the correlation between abnormal rsFCs and the level of suicide risk (assessed using the Nurses' Global Assessment of Suicide Risk, NGASR) in SA patients. Then, we applied support vector machines (SVMs) to classify SA vs. NSA in BD-II patients and predicted the risk of suicidality. SA patients showed significantly decreased frontolimbic rsFCs compared to NSA patients. The left amygdala-right middle frontal gyrus (orbital part) rsFC was negatively correlated with NGASR in the SA group, but not the severity of depressive or anxiety symptoms. Using frontolimbic rsFCs as features, the SVMs obtained an overall 84% classification accuracy in distinguishing SA and NSA. A significant correlation was observed between the SVMs-predicted NGASR and clinical assessed NGASR (*r* = 0.51, *p* = 0.001). Our results demonstrated that decreased rsFCs in the frontolimbic system might be critical objective features of suicidality in BD-II patients, and could be useful for objective prediction of suicidality risk in individuals.

## Introduction

Bipolar II disorder (BD-II), characterized by at least one episode of hypomania, one episode of major depression, and no history of a full manic episode, constitutes one of the major causes of suicide attempts (SA), especially during major depressive episodes (1, 2). A recent meta-analysis found that 32.4% of BD-II patients retrospectively reported a lifetime history of suicide attempts, leading to devastating social consequences (3). Some epidemiological studies demonstrated that suicide incidence and lethality in patients with BD-II were higher than with bipolar I disorder (2–7). Numerous studies have reported clinical features, including a family history of suicide, presence of hopelessness, loss of interest or pleasure, prior suicide attempts, and others, were associated with high suicide risk in individuals with bipolar disorder and might help identify at-risk patients (8, 9). Currently, clinical assessments such as the Nurses' Global Assessment of Suicide Risk (NGASR) (10) remain the cornerstone of suicide risk assessment for psychosis patients. However, clinical suicide risk evaluations primarily rely on retrospective information or self-reports by patients, which are inevitably subjective. Additionally, almost 80% of patients who have attempted suicide did not report their suicidal ideation to their doctors or healthcare providers (11). Therefore, it is challenging to accurately assess the risk of suicidality in patients with BD-II during depressive episodes in clinical practice, highlighting the need to identify reliable and accurate biomarkers for suicidality.

Functional magnetic resonance imaging (fMRI) is a noninvasive and repeatable brain neuroimaging technique that could be used to identify the potential neural circuits or networks associated with suicide attempts *in vivo* (11–13). Recent fMRI studies have shown that psychiatric disorders with suicide attempts are linked to structural and functional abnormalities in distinct brain networks, especially the frontolimbic system (14). It has been suggested that the frontolimbic system is heavily involved in mood regulation, including anxiety, reactive aggression, impulsivity, and decision-making, and may constitute a major component of the diathesis of suicidality (15–20). In particular, the amygdala plays critical roles in emotional reactions, decision-making, and memory processing, all of which may be related to suicide (21, 22). The frontal-amygdala system has been implicated in suicidal behaviors, possibly due to the ventral prefrontal cortex's inhibitory regulation impairment of the anterior limbic network (23–25). Although previous studies have demonstrated that abnormalities in the frontolimbic system might be one of the underlying neuropathological mechanisms of suicidal behavior, little evidence has been reported in BD-II patients with suicidal behavior. Moreover, it remains doubtful whether changes in functional connectivity in the frontolimbic system in BD-II patients with suicide attempts can be used to predict suicidality severity reliably.

To improve suicide prevention in clinical practice, it is essential to move from subjective classifications toward more objective approaches when evaluating the risk of suicidality. Sophisticated evidence-based methods utilizing neuroimaging measures can be useful in the early identification of suicide attempters and objective assessment of suicide risk (26). In contrast to traditional statistical methods, machine learning techniques allow predictions at the individual level, which will advance the successful application of neuroimaging in clinical medicine (11, 27–29). Although several recent studies used machine learning techniques based on neuroimaging characteristics to help with the early identification of mental disorders, including major depressive disorder and bipolar disorder (30, 31), few reports have focused on suicidal behaviors. To our knowledge, only one study applied random forest algorithms combined with altered resting-state functional connectivity (rsFC) and structural magnetic resonance imaging features grounded in the neurobiology of suicide to classify suicidal behavior in psychiatric inpatients (11). This previous study reported that neuroimaging biomarkers could be used to reliably classify suicidal behavior with 75% accuracy (sensitivity = 79.4%, specificity = 72.3%) (11). Also, no studies using machine learning to classify suicidal behavior with neuroimaging in BD-II patients have been reported.

In the present study, we recruited a cohort of participants, including 43 BD-II depression patients with at least one suicide attempt (SA) during a current depressive episode, 62 BD-II depression patients without a history of suicide attempts, and 64 HCs. Our goals were 2-fold. First, we aimed to examine alterations in rsFC between the bilateral amygdala and other frontolimbic regions in BD-II depression patients with SA. Second, we explored the feasibility of using machine learning techniques based on imaging features as a reliable biomarker for early identification of suicide attempters and predict suicide risk in BD-II patients currently experiencing a major depressive episode.

## Materials and Methods

### Participants

A total of 105 inpatients admitted to the Affiliated Brain Hospital of Nanjing Medical University from September 2014 to December 2017, and 64 healthy controls (HCs) were recruited. All participants were right-handed and native Han Chinese. Patients met the primary diagnosis criteria of Bipolar II Disorder and were currently experiencing a major depressive episode. The diagnosis of BD-II was determined based on the criteria of the Diagnostic and Statistical Manual of Mental Disorders, Fourth Edition, Text Revision (DSM-IV-TR), using the Mini-International Neuropsychiatric Interview (MINI, Chinese version) (32). The diagnosis was established by at least two board-certified psychiatrists with extensive expertise in affective disorders. The inclusion criteria were as follows. (1) The 17-item Hamilton Depression Rating Scale (HAMD-17) score was higher than 17 (33). (2) The patient's age was between 18 and 55 years. (3) No comorbidity with other DSM-IV axis-I disorders were indicated. (4) The patient did not undergo physical therapy or psychotherapy in the previous 6 months. (5) No psychotropic medications, including antidepressants, antipsychotics, mood stabilizers, and benzodiazepines, were taken in the previous 6 weeks. The following exclusion criteria were used. (1) The patient had a serious medical condition, such as an organic brain disorder or severe somatic disease, as confirmed by the patient's medical history or laboratory analysis. (2) The patient was pregnant or actively breastfeeding. (3) The patient was unable to undergo MRI scans. HCs from the local community did not exhibit any lifetime psychiatric disorders, history of substance abuse or dependence determined by the MINI, or history of neurological disorders. Similar exclusion criteria were used for the HCs. The first-degree relatives of the HCs were assessed using the Family History Screen for Epidemiological Studies to ensure they had no history of psychiatric illness (34).

The BD-II patients were divided into two subgroups. Forty-three individuals with at least one suicide attempt during a current major depressive episode were included in the SA group and 62 patients with no prior history of suicide attempts during the current or previous depressive episodes (NSA group). According to the American Psychiatric Association (2003), a suicide attempt was defined as at least one documented self-injurious act committed with the intent to die in the current major depressive episode, which was confirmed by medical records, and the HAMD-17 three-item (suicide) score was ≥2 (35, 36). Fifteen patients were excluded from the study due to excessive head movements (SA: *N* = 2; NSA: *N* = 3), demyelinating encephalopathy (SA: *N* = 1), brain atrophy (SA: *N* = 1), intracranial cyst (SA: *N* = 1; NSA: *N* = 3), lacunar infarction (NSA: *N* = 3), and poor image quality (SA: *N* = 1). Two HCs were excluded due to excessive head movements. 37 SA (age, 28.51 ± 8.95 years; 10M/27F), 53 NSA (30.75 ± 10.11, 20M/33F), and 62 HC (32.82 ± 9.84, 28M/34F) subjects were included in the final analyses. Each fMRI scan was conducted within 2 days of the clinical examination and prior to initiation of treatment.

### Assessment of Clinical Characteristics

The risk of suicidality in BD-II depression patients was defined as the total score based on the Nurses' Global Assessment of Suicide Risk (NGASR) (10). The NGASR is an “evidence-based” tool for suicide risk assessment based on both proximal factors (such as current mood state) and distal clinical factors (such as a family history of serious psychiatric problems or suicide). Given the complexity of suicide, 15 factors with different weights were included in the NGASR. The total NGASR score represents a numerical estimation of the level of suicide risk, with a maximum total score of 25. Thus, a higher score corresponds to a higher risk for suicide (see Supplementary Table 1). Additionally, the severity of the current depressive symptoms was evaluated using the 17-item HAMD (33), and the severity of the current anxiety symptoms was assessed using the Hamilton Anxiety Rating Scale (HAMA) (37). Other clinical features possibly associated with BD suicide risk also were assessed, including a rapid cycling type, disease course, age of onset, the polarity of the first episode, combination with a somatic disorder, accompanying psychotic characteristics, number of previous episodes of depression and hypomania, family history, occupation status, marital status, and years of education. The demographic and clinical characteristics of the participants are summarized in Table 1.

**Table 1:** Demographic and clinical characteristics of participants.

**Characteristics**	**SA**	**NSA**	**HC**	***t*/*F*/*x*^**2**^**	***p***
Numbers of subjects	37	53	62		—
Age, mean (SD), year	28.51 (8.95)	30.75 (10.11)	32.82 (9.84)	2.29^a^	0.105
Education, mean (SD), year	13.73 (3.29)	14.26 (2.85)	15.36 (2.73)	4.07^a^	0.019^*^
Course of disease mean (SD), month	74.70 (69.95)	66.94 (66.42)		0.53^b^	0.595
Gender, male/female	10M/27F	20M/33F	28M/34F	3.48^c^	0.175
marital status (Y/N)	13/24	23/30	34/28	3.51^c^	0.173
Seasonal characteristics(Y/N)	2/35	1/53			—
Age of onset mean (SD), year	22.24 (8.34)	25.00 (9.76)		−1.40^c^	0.166
Family history of mental disorder, Y/N	13/24	21/32		0.19^c^	0.666
Family history of suicide, Y/N	0/37	3/50			—
Polarity of first episode (depression/hypomania)	32/5	39/14		2.18^c^	0.140
Scores of HAMD-17	22.54 (3.60)	21.39 (2.71)		1.61^b^	0.114
Scores of HAMD-16 (without 3, suicide)	19.40 (3.36)	19.45 (2.48)		−0.08^b^	0.939
Scores of HAMA	14.91 (6.21)	17.39 (8.23)		−1.47^b^	0.147
Combined somatic disorder (Y/N)	11/26	14/39		0.12^c^	0.730
Psychotic characteristics (Y/N)	6/31	7/46		0.16^c^	0.690
Occupation (Y/N)	27/10	39/14		0.00^c^	0.948
Number of episodes of depression	3.51 (2.78)	2.92 (1.72)		1.24^b^	0.217
Number of episodes of hypomania	2.30 (2.83)	2.19 (1.91)		0.22^b^	0.828
Rapid cycling (Y/N)	11/26	13/40		0.30^c^	0.583
Comorbid substance abuse/dependence	6/31	3/50			—
Total score of NGASR	12.62 (2.96)	7.30 (3.08)		8.20^b^	0.000^***^

*HAMD-17, 17-item Hamilton Depression Rating Scale; HAMA, Hamilton Anxiety Rating Scale; NGASR, Nurses' Global Assessment of Suicide Risk*.

a *Univariate ANOVA*.

b *Two-sample t test*.

c *Pearson Chi-square test*.

* p < 0.05;

*** *p < 0.001*.

### Ethical Statement

This study abided by the ethical guidelines of the World Medical Association Declaration of Helsinki (38) and was approved by the Local Medical Ethics Committee of the Affiliated Brain Hospital of Nanjing Medical University (protocol number 2014-KY045). All participants were provided and signed a written informed consent form and were compensated financially for their participation.

### MRI Data Acquisition and Preprocessing

All participants underwent a resting-state scan using a 3.0T Siemens MRI system (Siemens Medical Solutions, Germany). They were instructed to keep their eyes closed, not to fall asleep, and to minimize movement. No participants were reported to have fallen asleep during scanning. Functional images were acquired with the following parameters: repetition time (TR)/echo time (TE) = 3,000 ms/40 ms; flip angle (FA) = 90°; field of view (FOV) = 240 × 240 mm^2^; matrix = 64 × 64; thickness/gap = 4.0 mm/0 mm; and slice number = 32. The recording session lasted 6 min and 45 s (133 volumes). The T1-weighted anatomical images were obtained using gradient-echo sequence (TR/TE/FA = 1,900 ms/2.48 ms/9°; slice number = 176; slice thickness = 1 mm; FOV = 250 × 250 mm^2^; and matrix size = 256 × 256). The data acquisition lasted 4 min and 18 s.

T1 images checked manually by an experienced neurologist and a radiologist for quality control. Functional images were preprocessed using the Data Processing Assistant for Resting-State fMRI (DPARSF; http://www.restfmri.net/forum/DPARSF) (39) and the Statistical Parametric Mapping software (SPM8; http://www.fil.ion.ucl.ac.uk/spm). To allow participants to adapt to the machine noise, the first six functional volumes were excluded for signal equilibrium. The remaining functional images underwent slice-timing and realignment. The functional volumes were normalized into the Montreal Neurological Institute (MNI) space with 3 × 3 × 3 mm^3^ resolution. Then functional images were smoothed with 6 mm full-width at half-maximum Gaussian kernel, linear detrending, and band-pass filtered at 0.01–0.08 Hz to reduce the effects of low-frequency drift and high-frequency noise. Participants who exhibited excessive head motion (>2.0 mm translation and/or 2.0° rotation) were excluded from further analysis. Finally, linear regression modeling was applied to control nuisance covariates, including head motion (Friston 24-parameter) (40), and signals of the whole brain, white matter and cerebrospinal fluid. The head motion was measured using frame-wise displacement (FD) and was not significantly different among the three groups (one-way ANOVA, *F* = 0.225, *p* = 0.80).

### Seed-Based Functional Connectivity Estimation

Since we specifically focused on the frontolimbic circuit, the bilateral amygdala was selected as the seed region due to its strong implications within the frontolimbic system for suicide behavior (21, 22, 41). Furthermore, 36 regions of interest (ROIs) were chosen to represent the frontolimbic system (including the bilateral amygdala) based on automated anatomical labeling (see Supplementary Table 2). Amygdala-seed based functional connectivity calculations were conducted using the resting-state fMRI data analysis Toolkit (REST; http://www.restfmri.net) (42). Briefly, the time series were extracted by averaging the signals of all the voxels within the atlas regions. Then Pearson correlation coefficients were computed using the full-length time-series for each of the paired regions for each subject. Subsequently, the correlation coefficients were converted to *z*-scores using Fisher-Z transformation for future analysis.

### Statistical Analysis

#### Demographic and Clinical Characteristics

For statistics among the three groups, χ^2^ tests were used to compare gender and marital status, and one-way analysis of variance (ANOVA) was used to analyze age and education. When comparing within the SA and NSA groups, χ^2^ tests were conducted to compare family history, the polarity of the first episode, combination with a mental or somatic disorder, accompanying psychotic characteristics, and rapid cycling type. Two-sample *t*-tests were used to compare the course of illness, age of onset, HAMD-17, HAMD-16 (without three-item, suicide), HAMA, number of previous episodes, and NGASR. A *p*-value < 0.05 was considered to be statistically significant.

#### Resting-State Functional Connectivity Comparisons

We compared the bilateral amygdala-seed-based rsFC (*z* scores) for the frontolimbic system among the three groups using a one-way ANCOVA, with age, education, and mean FD as covariates. Significance was determined after FDR correction with respect to the number of estimated rsFCs (*N* = 70) at *p* < 0.05. Then, *post-hoc* comparisons were performed between the groups (SA vs. NSA, SA vs. HC, and NSA vs. HC), and significance was FDR corrected based on the number of groups (*N* = 3) at *p* < 0.05.

#### Correlation Analysis Between Resting-State Functional Connectivity and Clinical Assessments

Among the rsFCs in the frontolimbic system that showed significant differences between the SA and NSA groups, we computed the partial correlation to assess the association between the rsFCs and suicide risk. The suicide risk was measured as the total NGASR score while controlling for potential confounding factors (e.g., age, education, mean FD, and severity of depressive symptoms) in the SA group. The significance was FDR-corrected for the number of significantly different rsFCs between the SA and NSA groups (*N* = 7). Then, additional control Spearman correlation analyses were carried out to examine the associations between the FCs that were significantly correlated with suicide risk in the first step, and the severity of depressive symptoms (measured as the total score of HAMD-16, without the suicide-item), as well as anxiety symptoms (measured as the total HAMA score) in the SA group, respectively.

#### Support Vector Machine Classification and Regression

Support vector machines (SVMs) are popular supervised learning models for classification and regression problems that construct a hyperplane or set of hyperplanes in a high- or infinite-dimensional space (43). We applied SVMs to classify SA vs. NSA patients and predict suicide risk. The included features were selected from the significantly different frontolimbic rsFCs between the SA and NSA groups for classification analysis. Moreover, the radial basis function (RBF) SVM with a Gaussian kernel was used, and 5-fold cross-validation was conducted. The 5-fold cross-validation approach randomly divided all patients into five equal subsets. Of the five subsets, four subsets served as training data to train an optimal model, and the remaining subset was used to test the performance of the model. This procedure was repeated five times until each subset was used once as a testing subset. The average of the results from all repetitions was considered as the final performance. To test the robustness of the classifier, permutation tests were repeated 1,000 times, which provided a reference distribution. For each permutation run, we randomly shuffled the group labels and followed the proposed classification model. The *p*-value was obtained by comparing the observed actual classification performances to the reference distribution.

The significantly associated frontolimbic FCs from correlation analysis were included for support vector regression (SVR). The performance of the SVR model was evaluated using the Spearman correlation between the model estimation and the clinical assessment.

## Results

### Demographic and Clinical Characteristics

The demographic and clinical characteristics of the participants are summarized in Table 1. There were no statistical differences in age, gender, and marital status among the three groups. However, a significant difference was observed in years of education (*F*_2, 149_ = 4.07, *p* = 0.019). For comparisons between the SA and NSA groups, we did not observe any significant differences in years of education, duration of disease, age of BD onset, family history of mental disorder, the polarity of the first episode, HAMD-17, HAMD-16 (without three-item, suicide), HAMA, combination with a somatic disorder, psychotic characteristics, number of previous episodes of depression and hypomania, occupation status, and rapid cycling. The NGASR score was significantly increased in the SA group compared to the NSA group [*t*_(1, 88)_ = 8.2, *p* < 0.001].

### Decreased Frontolimbic rsFC in the SA Group Compared to the NSA Group

**Figure 1:**
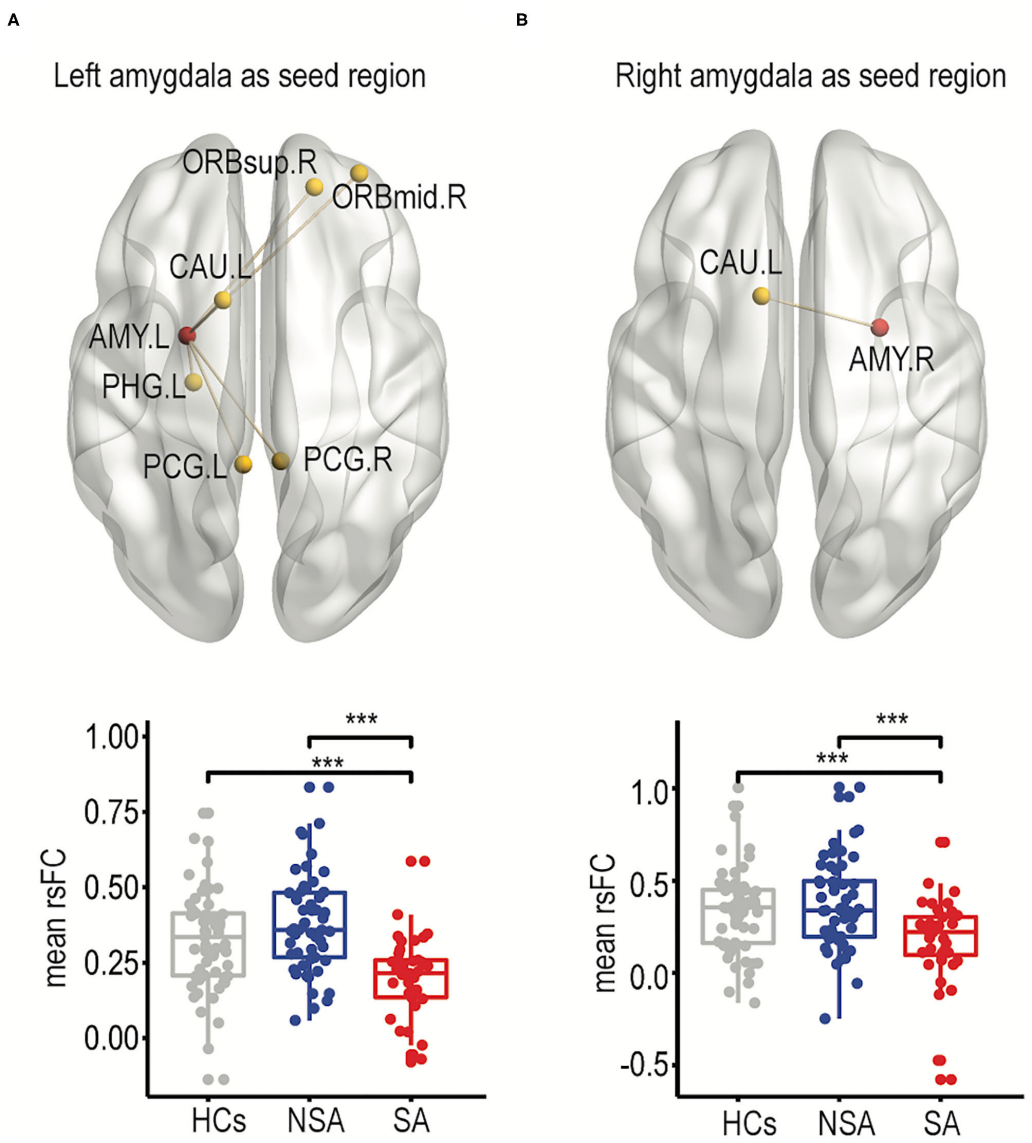
Frontolimbic rsFCs comparisons between SA, NSA, and HCs. The significantly different resting-state functional connectivity between suicide attempters and non-suicide attempters was shown for the left amygdala as the seed region **(A)** and the right amygdala as the seed region **(B)**. In addition, the mean FCs among three groups were shown as box plotted as well (right panel). The significances of all comparisons were FDR corrected. ***p < 0.001. HC, healthy controls; NSA, bipolar II disorder depression patients without suicidal attempt; SA, bipolar II disorder depression patients with suicide attempt; AMY.L, left amygdala; AMY.R, right amygdala; ORBsup.R, right superior frontal gyrus (dorsolateral); ORBmid.R, right middle frontal gyrus (orbital part); PCG.L, left posterior cingulate gyrus; PCG.R, right posterior cingulate gyrus; PHG.L, left parahippocampal gyrus; CAU.L, left caudate nucleus.

The rsFCs between the bilateral amygdala and other frontolimbic regions were compared among the three groups using a one-way ANCOVA. Specifically, one-way ANCOVAs comparing rsFCs between the left or right amygdala as the seed region and other frontolimbic regions were carried out and included age, education, and mean FD as covariates. When using the left amygdala as the seed region, there were significantly different rsFCs between the right superior frontal gyrus (dorsolateral), right middle frontal gyrus (orbital part), bilateral posterior cingulate gyrus, left parahippocampal gyrus, and left caudate nucleus among the SA, NSA, and HC groups after FDR correction (see Figure 1A, Supplementary Table 3). Furthermore, the *post-hoc* test showed that, compared to the NSA group, rsFCs were significantly decreased in the SA group [*t*_(1, 88)_ = 5.58, *p* < 0.001; see Figure 1A, Supplementary Table 4].

When using the right amygdala as the seed region, only rsFCs in the left caudate were significantly different among the SA, NSA, and HC groups after FDR correction (for details, see Figure 1B, Supplementary Table 3). The *post-hoc* test showed that the SA group exhibited significantly decreased connectivity in the left caudate compared to the NSA group [*t*_(1, 88)_ = 3.83, *p* < 0.001; see Figure 1B, Supplementary Table 4].

### Correlation Analysis Between the Resting-State Functional Connectivity and Clinical Assessments

**Figure 2:**
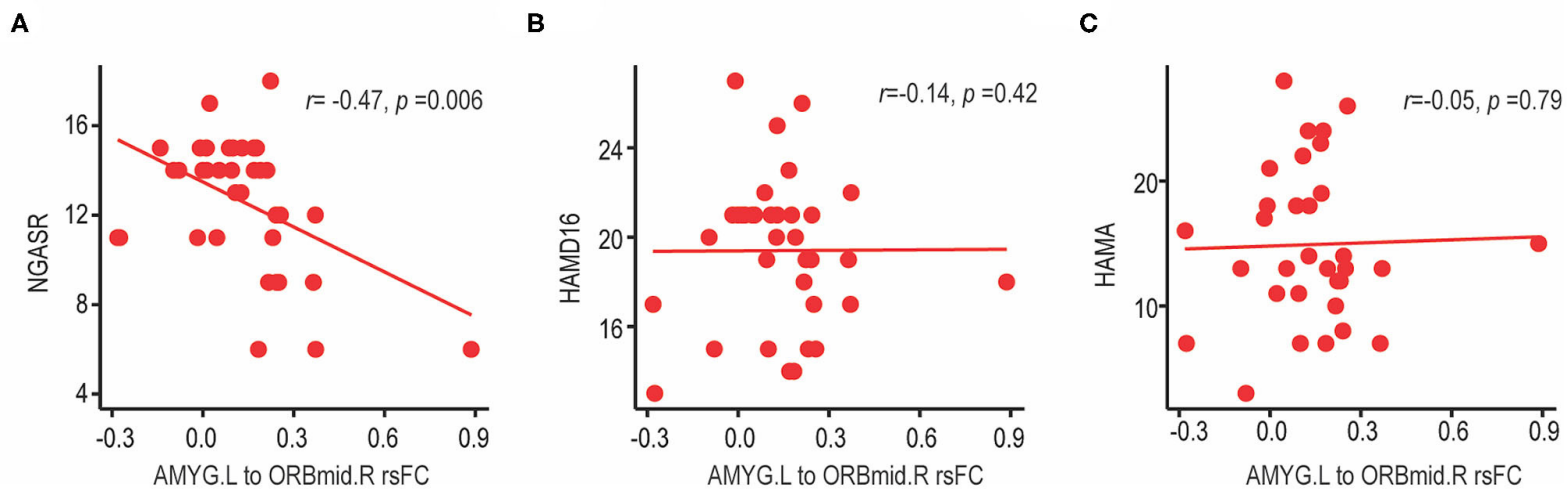
Correlation between left amygdala-right middle frontal gyrus (orbital part) rsFC and clinical characteristics in BD-II depression patients with suicide attempts. **(A)** The left amygdala-right middle frontal gyrus (orbital part) rsFC showed a significant negative correlation with suicide risk (measured by NGASR score). FCs were Fisher z-transformed and relationship was analyzed using partial correlation controlling for age, education, mean FD factors and HAMD-16 score (without three-item, suicide). **(B)** There was no association between the left amygdala-right middle frontal gyrus (orbital part) rsFC and the severity of depressive symptoms (measured by HAMD-16 scores, without suicide-item), or **(C)** severity of anxiety symptoms (measured by HAMA score). NGASR, Nurses' Global Assessment of Suicide Risk; HAMD, Hamilton Depression Rating Scale; HAMA, Hamilton Anxiety Rating Scale; AMY.L, left amygdala; ORBmid.R, right middle frontal gyrus (orbital part).

After identifying the significantly altered frontolimbic rsFCs (Figure 1), correlations between the altered rsFCs and the risk of suicide assessed by NGASR in the SA group were analyzed using a partial correlation that controlled for age, education, mean FD factors, and the HAMD-16 score. We observed a significant negative correlation between the left amygdala-right middle frontal gyrus (orbital part) rsFC and the NGASR (see Figure 2A, *r* = −0.47, *p* = 0.006, FDR corrected). This result indicated that the weaker the rsFC between the left amygdala and right middle frontal gyrus (orbital part), the higher the risk of suicidality during a current major depressive episode. There was no association between the left amygdala to right middle frontal gyrus (orbital part) and the HAMD-16 score (see Figure 2B, *r*_*s*_= −0.14, *p* = 0.42) or the HAMA score (see Figure 2C, *r*_*s*_ = −0.05, *p* = 0.79), suggesting that decreased frontolimbic rsFCs were specific to suicide risk but not related to the severity of the depressive or anxiety symptoms. Note that our findings were not driven by the one potential outlier because the resultss maintained when the outlier was excluded (Supplementary Figure 1).

### SA and NSA SVM Classification Performance

**Figure 3:**
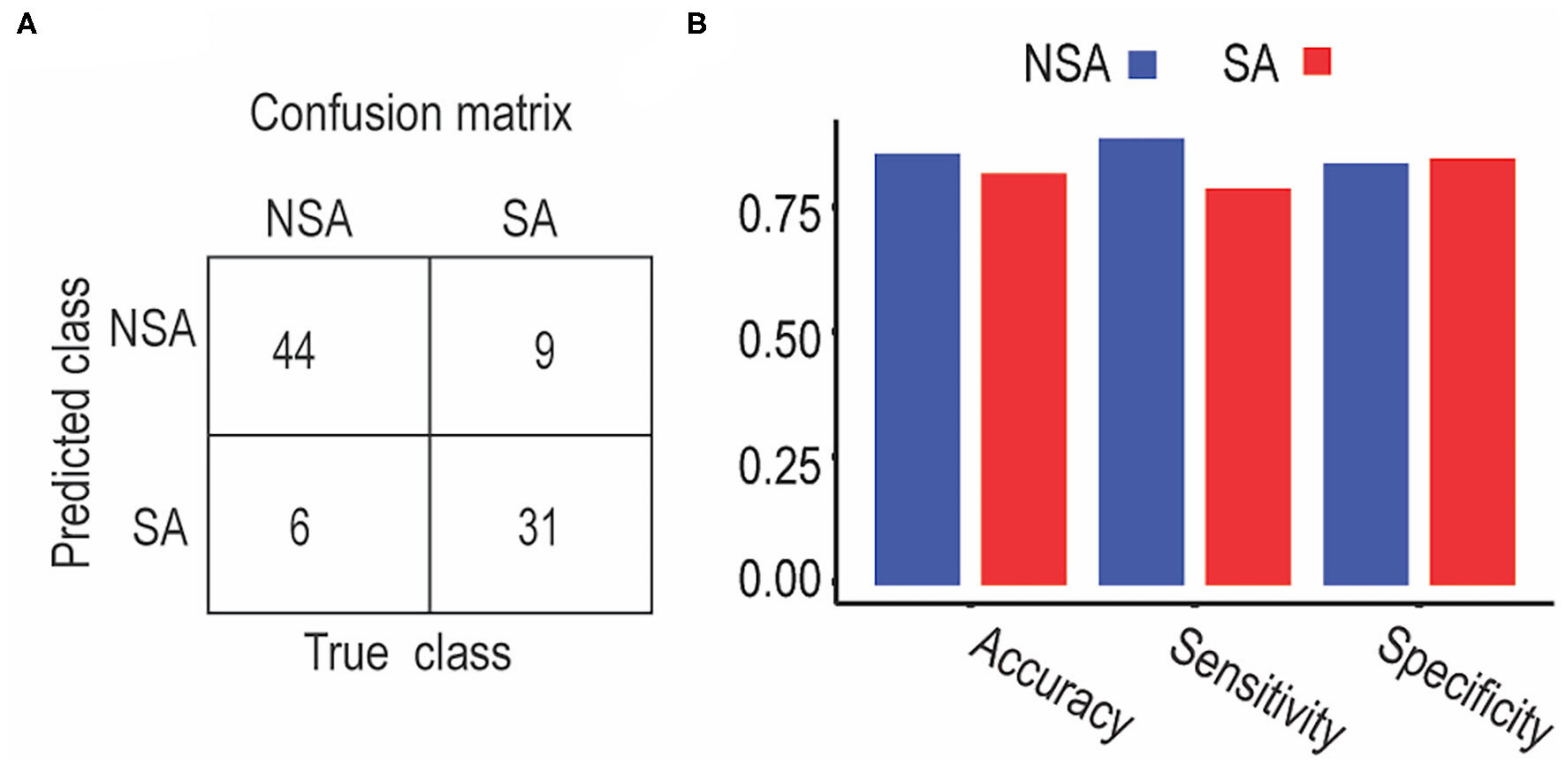
Classification performance between SA and NSA patients using support vector machine. **(A)** Classification confusion matrices. Each row of the matrix represents the occurrences in an actual class, while each column represents the occurrences in a predicted class. **(B)** Classification accuracy, sensitivity, and specificity for NSA and SA, respectively.

We used the altered frontolimbic rsFCs as features and trained SVM models to discriminate between suicide attempters and non-suicide attempters. Figure 3A shows the confusion matrices for the proposed SVM models. The diagonal elements of the confusion matrices represented the instances for which the predicted class was equal to the actual class. The off-diagonal elements indicated the instances that were mislabeled by the classifier. Sensitivity, specificity, and accuracy were calculated based on the confusion matrices as follows: sensitivity = TP/TP + FN; specificity = TN/TN + FP; accuracy = (sensitivity + specificity)/2, where TP was the number of true-positive samples, and FN was the number of false-negative samples, TN was the number of true negative samples, and FP was the number of false-positive samples. The SVM approach exhibited an overall classification accuracy of 84%. The classification performances for the NSA group (accuracy: 85%; sensitivity: 88%; specificity: 83%) and the SA group (accuracy: 81%; sensitivity: 78%; specificity: 84%) are illustrated in Figure 3B. Permutation tests showed that the average accuracy expected due to random shuffling was approximately 69%, and the performance of the overall classification accuracy was statistically significant at *p* < 0.01.

### Suicide Risk Prediction Using Support Vector Regression in SA Patients

**Figure 4:**
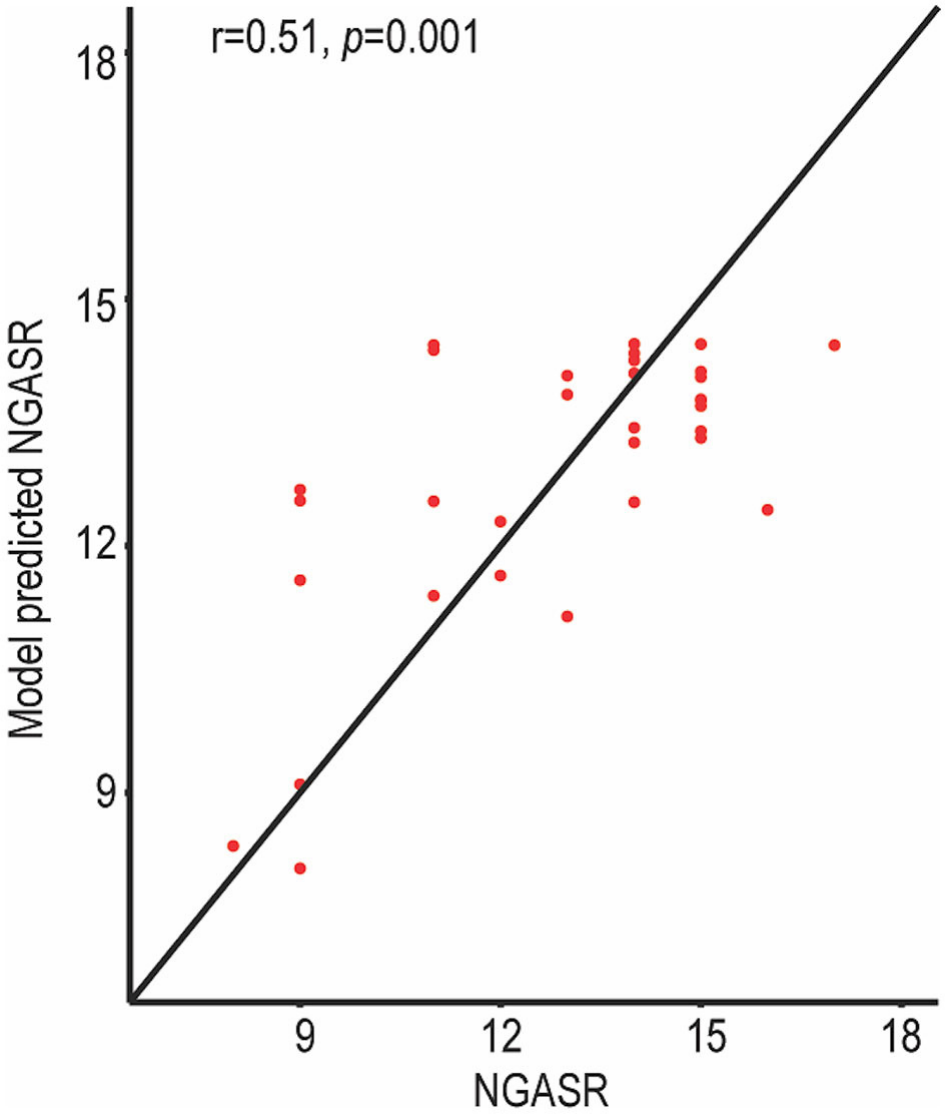
NGASR values estimated using the support vector regression model in SA patients. The predictive performance was evaluated by Spearman correlation between the predicted NGASR values and professionally evaluated NGASR values. The correlation between the predicted NGASR values and the professional NGASR values was significant.

A support vector regression model was used to predict individual suicidal risk in the SA group (Figure 4). The identified left amygdala to right middle frontal gyrus (orbital part) rsFC (Figure 2) were used as inputs for the support vector regression model. To evaluate the support vector regression model, we used 5-fold cross-validation. Figure 4 illustrates the predictive performance for the support vector regression model, based on the altered frontolimbic rsFCs. The diagonal line represents an exact match between the subjective NGASR values estimated by clinical experts and the objective NGASR values as estimated by our model. Above the diagonal line, the objective NGASR values were higher than the subjective NGASR values and vice versa for values below the diagonal line. Importantly, the correlation between the predicted NGASR values and the subjective NGASR values was significant (*r* = 0.51, *p* = 0.001), indicating the capability of our regression model to predict individual suicidality risk accurately.

## Discussion

In the current study, we contributed to the identification of neuroimaging biomarkers for suicide attempts in patients with BD-II depression. We also built a machine model to distinguish suicide attempters from non-suicide attempters and provided an objective estimation of suicidality risk based on neuroimaging characteristics at the individual level. Specifically, we found that the SA group exhibited significantly decreased frontolimbic rsFCs compared to the NSA group. Also, the rsFC between the left amygdala and the right middle frontal gyrus (orbital part) was negatively correlated with a current risk of suicidality in the SA group, as assessed with the NGASR. Furthermore, suicide attempters were identified with high accuracy (83.78%) at the individual level based on altered frontolimbic rsFCs in the SVM model, and the SVMs-predicted risk of suicide was significantly correlated with the clinical assessment. Our results indicated that the decreased frontolimbic rsFCs might be a critical biomarker of suicidality in patients with BD-II and currently experiencing a major depressive episode. One interpretation could be that alterations in the frontolimbic system impaired mood regulation and decision-making, which predisposed depressed BD-II patients to act more impulsively and attempt suicide.

Patients with suicidal behaviors typically show impaired goal-directed behaviors, judgment, and decision-making, which have been suggested to result from decreased modulation of the frontal cortex-limbic system (44, 45). It has been reported that decision-making is an emotionally dependent process in which both the orbitofrontal cortex (OFC) and the amygdala are involved (46, 47). Patients with damages in the OFC and amygdala often exhibit impulsive patterns of behavior (46). Previous MRI studies have confirmed abnormal structural changes, including decreased OFC volume and increased amygdala volume in SA patients (35, 48). Also, decreased white matter volume in the OFC in bipolar disorder patients with suicidality (49) and lower anisotropy between the left orbitofrontal area and the left anterior-limbic system have been reported. These data indicated that structural impairments in the frontolimbic system were linked to suicidal behavior in patients with bipolar disorder (50). On the other hand, the OFC plays an essential role in decision-making and evaluating possible outcomes through inhibition of the amygdala. Lower levels of inhibition of the amygdala are associated with higher rates of suicide attempts and higher mortality (51). An fMRI study found that SA patients with bipolar disorder showed decreased FCs in the left ventral prefrontal and right rostral prefrontal regions when the amygdala was selected as the seed region during the processing of happy and neutral faces (41). Similar to previous reports, we demonstrated hypo-connectivity between the left amygdala and right middle frontal gyrus (orbital part) in BD-II depression patients with suicide attempts compared to non-suicide attempters. These findings further supported that the dysfunction of the frontolimbic system could be a potential neuropathological basis for suicidal behavior in BD-II depression patients, regardless of the severity of the depressive episode.

The current study showed significantly decreased frontolimbic FCs, both in inter-hemisphere (such as left amygdala to right dorsolateral superior frontal gyrus, right orbital middle frontal gyrus, and right posterior cingulate gyrus, and right amygdala to left caudate) and intra-hemisphere (such as left amygdala to left parahippocampal gyrus and left caudate) assessments. This result was consistent with recent diffusion tensor imaging (DTI) studies, which demonstrated that lesions in the bilateral uncinate fasciculus (UF) and corpus callosum (CC) were related to suicide attempts in bipolar disorder patients (24, 52, 53). The corpus callosum (CC) is the largest connectivity pathway and allows information to pass between the two cerebral hemispheres. The uncinated fasciculus (UF) connects the inter-hemispheric parts of the limbic system, such as the amygdala, with frontal regions such as the orbitofrontal cortex. Structural abnormalities of the CC and bilateral UF suggested that abnormal FC of frontolimbic systems in BD-II depression patients with SA might also be involved in the inter-hemisphere and intra-hemisphere regions. Nevertheless, the asymmetry and lateralized functional changes of frontolimbic systems related to attempted suicide in BD-II depression patients seen in this study require further investigation.

The SA group in this study also showed decreased connectivity within other frontolimbic regions compared to the NSA group, including the left amygdala to right superior frontal gyrus (dorsolateral), bilateral posterior cingulate gyrus, left parahippocampal gyrus, and left caudate, as well as the right amygdala to left caudate. Alterations in the dorsolateral prefrontal cortex result in impaired executive functions, including top-down regulation of goal-direct behaviors, control of impulsive behavior, and decision-making, which are related to attempted suicide behavior (54–56). Recent structural MRI researches have demonstrated reductions in gray matter volume (GMV) in the dorsolateral prefrontal cortex in SA patients with bipolar disorder (54, 56–58). The parahippocampal gyrus is highly connected with the amygdala (59), and decreased rsFC between the parahippocampal gyrus and the amygdala has been associated with dysphoria (60), which could increase the risk for suicide. Moreover, insufficient connectivity between the amygdala and parahippocampal gyrus during the resting state could be a compensatory process to counteract hyper-connectivity during the processing of negative emotional memories (41), which was an interpretation of the reduced rsFC associated with suicide risk. Also, abnormalities within the cingulate cortex were confirmed to be associated with suicidal ideation and impulsivity (61). The caudate nucleus participates in reward processing and decision-making, which also is related to suicidal behavior (62). Overall, our findings reinforced that many amygdala-seed rsFCs in the frontolimbic regions were related to the attempted suicide behavior associated with BD-II depression.

Although neuroimaging studies on suicide behavior have seen significant advances, most studies have reported the results at the group level, making it challenging to apply the results in actual clinical settings (18, 20, 24, 56). Machine learning technologies can play a vital role in this aspect, as they can assess patterns in large data volumes and make individualized predictions (63, 64). One previous study combined a random forest algorithm and abnormal functional connectivity and reported that neural biomarkers might be useful to classify suicidal behavior in psychiatric inpatients (11). Our study has improved upon that study in several ways. There was no matched healthy control group in the previous study. Also, no multiple comparison correction was applied in the previous study to select neuroimaging features for the classification model, which could have resulted in the inclusions of false positives among the features that were used. To our knowledge, no previous investigations have included the suicide behavior of BD-II patients. Here, we demonstrated that decreased frontolimbic rsFCs could act as objective neuroimaging markers to help identify suicide attempters among BD-II depression patients using a support vector machine classifier with high accuracy and efficiency. The ability to classify suicide attempters through objective neuroimaging features presents a critical advance because patients at high risk of suicidality frequently conceal their suicide intentions prior to their suicide attempt (65). We also demonstrated the ability to predict suicide risk at the individual level. Future studies should integrate additional useful information from multiple domains, such as clinical assessments, genetics, electrophysiology, and neuropathology, to improve the discriminatory ability of attempted suicide behavior in the clinic.

Several limitations of this study should be noted. First, we could not distinguish whether an abnormal frontolimbic system rsFC in BD-II patients with a suicide attempt and currently in a major depressive were state-dependent markers or trait markers because of the cross-sectional design of the study. In the future, longitudinal follow-up studies across different mood phases will be needed to address this question. Second, although the use of the seed-based method increased the interpretability, our findings were limited to the selected regions. A distinct advantage of using a whole-brain study is to avoiding potential bias derived from focus on the chosen regions of interest. Finally, additional replication and validation in multiple centers with larger cohorts will be needed.

In summary, we demonstrated that the frontolimbic rsFCs in BD-II depression patients with a suicide attempt were decreased compared to non-suicide attempters, and decreased rsFCs were related to a higher risk of suicide. The integration of alterations in frontolimbic rsFCs and machine learning techniques helped identify suicide attempters and objectively assess the risk of suicidality at the individual level. Thus, an altered frontolimbic rsFC could be a reliable biomarker for suicide prevention and intervention in clinical practice. Overall, these findings provided new insights for early intervention and prevention strategies for suicide.

## Data Availability Statement

The raw data supporting the conclusions of this article will be made available by the authors, without undue reservation.

## Ethics Statement

The studies involving human participants were reviewed and approved by the Local Medical Ethics Committee of the Affiliated Brain Hospital of Nanjing Medical University. The patients/participants provided their written informed consent to participate in this study.

## Author Contributions

RZ formulated the research questions, designed the study, conducted the research, analyzed the data, and assisted in writing the article. STi, HW, HJ, XW, and JS analyzed the data and assisted in writing the article. QW designed the study and assisted in writing the article. RY, STa, and HL designed the study, conducted the research, and assisted in writing the article. QL and ZY formulated the research questions, designed the study, analyzed the data, and assisted in writing the article. All authors contributed to the article and approved the submitted version.

## Conflict of Interest

The authors declare that the research was conducted in the absence of any commercial or financial relationships that could be construed as a potential conflict of interest.
