# Supplementary material for: Discriminating Suicide Attempters and Predicting Suicide Risk Using Altered Frontolimbic Resting-State Functional Connectivity in Patients With Bipolar II Disorder
Source: Front Psychiatry. 2020 Nov 26;11:597770. doi: 10.3389/fpsyt.2020.597770 (PMC7725800; doi:10.3389/fpsyt.2020.597770)
Supplement: Supplementary file 1 [file Data_Sheet_1.docx]

**Discriminating Suicide Attempters and Predicting Suicide Risk Using Altered Frontolimbic Resting-state Functional Connectivity in Patients with Bipolar II Disorder**

***Supplementary Information***

Supplementary information: 1 Figure, 4 Tables


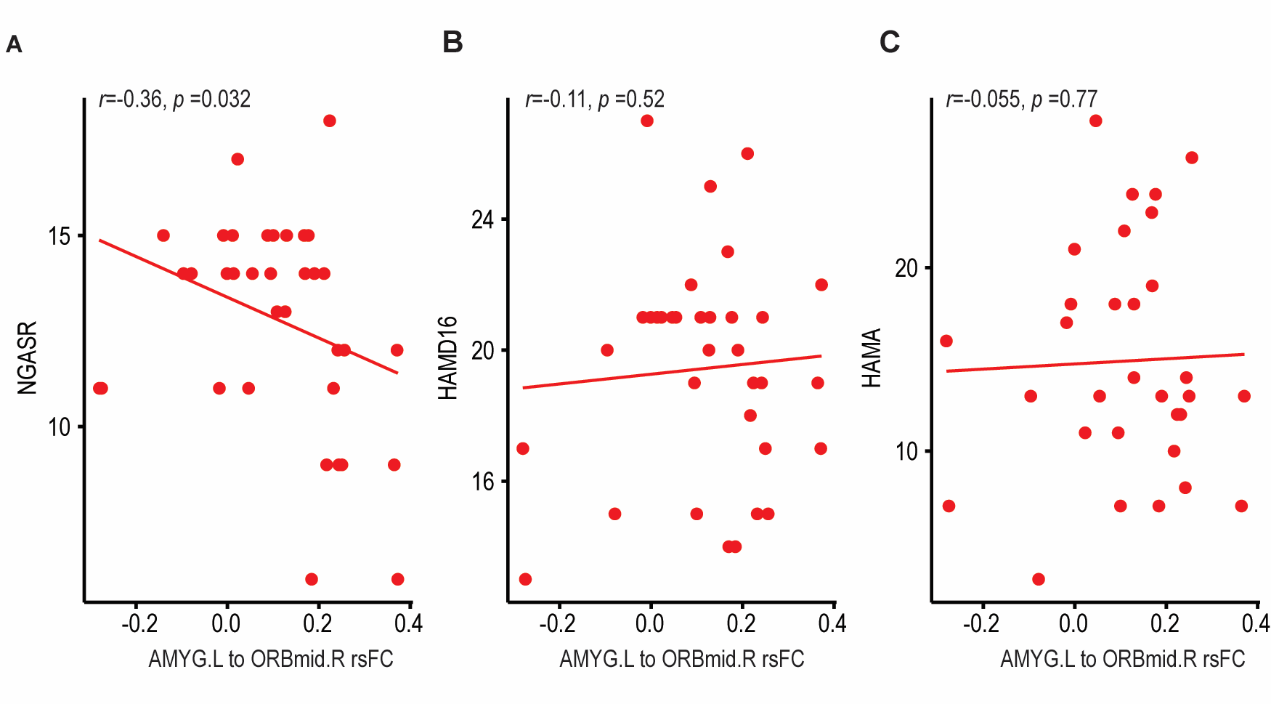


**Fig.S1** Correlation between left amygdala-right middle frontal gyrus (orbital part) rsFC and clinical characteristics in BD-II depression patients with suicide attempt without the outlier

**Table S1. The Nurses’ Global Assessment of Suicide Risk (NGASR)**

| **Predictor variable** | **Value** |
| --- | --- |
| Presence/influence of hopelessness | 3 |
| Recent stressful life event, for example, job loss, financial  worries, pending court action | 1 |
| Evidence of persecutory voices/beliefs | 1 |
| Evidence of depression/loss of interest or loss of pleasure | 3 |
| Evidence of withdrawal | 1 |
| Warning of suicidal intent | 1 |
| Evidence of a plan to commit suicide | 3 |
| Family history of serious psychiatric problems or suicide | 1 |
| Recent bereavement or relationship breakdown | 3 |
| History of psychosis | 1 |
| Widow/widower | 1 |
| Prior suicide attempt | 3 |
| History of socio-economic deprivation | 1 |
| History of alcohol and/or alcohol misuse | 1 |
| Presence of terminal illness | 1 |
| Total |  |

**Table S2. Regions of Interesting in Frontolimbic System from Automated Anatomical Labeling (ALL) Atlas**

| **Regions** | **abbr.** |
| --- | --- |
| Superior frontal gyrus, dorsolateral | SFGdor.L |
| Superior frontal gyrus, dorsolateral | SFGdor.R |
| Superior frontal gyrus, orbital part | ORBsup.L |
| Superior frontal gyrus, orbital part | ORBsup.R |
| Middle frontal gyrus | MFG.L |
| Middle frontal gyrus | MFG.R |
| Middle frontal gyrus, orbital part | ORBmid.L |
| Middle frontal gyrus, orbital part | ORBmid.R |
| Inferior frontal gyrus, opercular part | IFGoperc.L |
| Inferior frontal gyrus, opercular part | IFGoperc.R |
| Inferior frontal gyrus, triangular part | IFGtriang.L |
| Inferior frontal gyrus, triangular part | IFGtriang.R |
| Inferior frontal gyrus, orbital part | ORBinf.L |
| Inferior frontal gyrus, orbital part | ORBinf.R |
| Insula | INS.L |
| Insula | INS.R |
| Anterior cingulate and paracingulate gyri | ACG.L |
| Anterior cingulate and paracingulate gyri | ACG.R |
| Median cingulate and paracingulate gyri | DCG.L |
| Median cingulate and paracingulate gyri | DCG.R |
| Posterior cingulate gyrus | PCG.L |
| Posterior cingulate gyrus | PCG.R |
| Hippocampus | HIP.L |
| Hippocampus | HIP.R |
| Parahippocampal gyrus | PHG.L |
| Parahippocampal gyrus | PHG.R |
| Amygdala | AMYG.L |
| Amygdala | AMYG.R |
| Caudate nucleus | CAU.L |
| Caudate nucleus | CAU.R |
| Lenticular nucleus, putamen | PUT.L |
| Lenticular nucleus, putamen | PUT.R |
| Lenticular nucleus, pallidum | PAL.L |
| Lenticular nucleus, pallidum | PAL.R |
| Thalamus | THA.L |
| Thalamus | THA.R |

**Table S3. ANCOVA Analyses of Functional Connectivity with Left Amygdala as Seed**

| Seed region | Target regions | F | *P_FDR_* | Eta^2^ |
| --- | --- | --- | --- | --- |
| AMYG.L | SFGdor.R | 5.807 | 0.004^**^ | 0.074 |
|  | ORBmid.R | 5.959 | 0.003^**^ | 0.076 |
|  | PCG.L | 6.574 | 0.002^**^ | 0.083 |
|  | PCG.R | 6.000 | 0.003^**^ | 0.076 |
|  | PHG.L | 10.176 | <0.001^***^ | 0.123 |
|  | CAU.L | 5.995 | 0.003^**^ | 0.076 |
| AMYG.R | CAU.L | 7.757 | 0.001^**^ | 0.097 |

After FDR correction, there were still significantly decreased connectivity among SA, NSA and HCs, including left amygdala-right superior frontal gyrus (dorsolateral), left amygdala- right middle frontal gyrus (orbital part), left amygdala-bilateral posterior cingulate gyrus, left amygdala-left parahippocampal gyrus, left amygdala-left caudate and right amygdala-left caudate. Abbreviations: SA: BD-II depression patients with at least one suicide attempt during current major depressive disorder episode; NSA: BD-II depression patients without history of prior suicide attempts; HCs：health controls; AMYG.L: left amygdala; AMYG.R: right amygdala; SFGdor.R: right superior frontal gyrus (dorsolateral); ORBmid.R: right middle frontal gyrus (orbital part); PCG.L: left posterior cingulate gyrus; PCG.R: right posterior cingulate gyrus; PHG.L: left parahippocampal gyrus; CAU.L: left caudate nucleus.

**p*<0.05; ***p*<0.01; ****p*<0.001

**Table S4. Decreased Frontolimbic rsFC in SA compared to NSA after FDR Correction**

| Seed region | Target regions | Mean (SD) | 95% Confidence Interval | | *P_FDR_* |
| --- | --- | --- | --- | --- | --- |
| AMYG.L | SFGdor.R | -0.15(0.05) | -0.28 | -0.02 | 0.016^*^ |
|  | ORBmid.R | -0.17(0.05) | -0.28 | -0.05 | 0.002^**^ |
|  | PCG.L | -0.17(0.05) | -0.28 | -0.06 | 0.001^**^ |
|  | PCG.R | -0.17(0.05) | -0.28 | -0.05 | 0.002^**^ |
|  | PHG.L | -0.28(0.06) | -0.43 | -0.13 | <0.001^***^ |
|  | CAU.L | -0.16(0.05) | -0.29 | -0.04 | 0.006^**^ |
| AMYG.R | CAU.L | -0.20(0.05) | -0.32 | -0.07 | 0.001^**^ |

After FDR correction, patients in SA group still were demonstrated significantly decreased connectivity, including left amygdala-right dorsolateral superior frontal gyrus, left amygdala-right orbital middle frontal gyrus, left amygdala-bilateral posterior cingulate gyrus, left amygdala-left parahippocampal gyrus, left amygdala-left caudate and right amygdala-left caudate, compared to NSA group. Abbreviations: SA: BD-II depression patients with at least one suicide attempt during current major depressive disorder episode; NSA: BD-II depression patients without history of prior suicide attempts; rsFC: resting state functional connectivity; AMYG.L: left amygdala; AMYG.R: right amygdala; SFGdor.R: right superior frontal gyrus (dorsolateral); ORBmid.R: right middle frontal gyrus (orbital part); PCG.L: left posterior cingulate gyrus; PCG.R: right posterior cingulate gyrus; PHG.L: left parahippocampal gyrus; CAU.L: left caudate nucleus.

*：*p*<0.05；**: *p*<0.01；***：*p*<0.001.
